# An easy-to-use AIHF-nomogram to predict advanced liver fibrosis in patients with autoimmune hepatitis

**DOI:** 10.3389/fimmu.2023.1130362

**Published:** 2023-05-17

**Authors:** Zhiyi Zhang, Jian Wang, Huali Wang, Yuanwang Qiu, Li Zhu, Jiacheng Liu, Yun Chen, Yiguang Li, Yilin Liu, Yuxin Chen, Shengxia Yin, Xin Tong, Xiaomin Yan, Yali Xiong, Yongfeng Yang, Qun Zhang, Jie Li, Chuanwu Zhu, Chao Wu, Rui Huang

**Affiliations:** ^1^Department of Infectious Diseases, Nanjing Drum Tower Hospital Clinical College of Nanjing University of Chinese Medicine, Nanjing, China; ^2^Department of Infectious Diseases, Nanjing Drum Tower Hospital, Affiliated Hospital of Medical School, Nanjing University, Nanjing, China; ^3^Institute of Viruses and Infectious Diseases, Nanjing University, Nanjing, China; ^4^Department of General Practice, Nanjing Second Hospital, Nanjing University of Chinese Medicine, Nanjing, China; ^5^Department of Infectious Diseases, The Fifth People’s Hospital of Wuxi, Wuxi, China; ^6^Department of Infectious Diseases, The Affiliated Infectious Diseases Hospital of Soochow University, Suzhou, China; ^7^Department of Infectious Diseases, Nanjing Drum Tower Hospital Clinical College of Nanjing Medical University, Nanjing, China; ^8^Department of Laboratory Medicine, Nanjing Drum Tower Hospital, Affiliated Hospital of Medical School, Nanjing University, Nanjing, China; ^9^Department of Hepatology, Nanjing Second Hospital, Nanjing University of Chinese Medicine, Nanjing, China; ^10^Department of Infectious Diseases, Zhongda Hospital Southeast University, Nanjing, China

**Keywords:** autoimmune hepatitis, liver fibrosis, nomogram, non-invasive test, predictive model

## Abstract

**Background:**

The evaluation of liver fibrosis is essential in the management of patients with autoimmune hepatitis (AIH). We aimed to establish and validate an easy-to-use nomogram to identify AIH patients with advanced liver fibrosis.

**Methods:**

AIH patients who underwent liver biopsies were included and randomly divided into a training set and a validation set. The least absolute shrinkage and selection operator (LASSO) regression was used to select independent predictors of advanced liver fibrosis from the training set, which were utilized to establish a nomogram. The performance of the nomogram was evaluated using the receiver characteristic curve (ROC), calibration curve, and decision curve analysis (DCA).

**Results:**

The median age of 235 patients with AIH was 54 years old, with 83.0% of them being female. Six independent factors associated with advanced fibrosis, including sex, age, red cell distribution width, platelets, alkaline phosphatase, and prothrombin time, were combined to construct a predictive AIH fibrosis (AIHF)-nomogram. The AIHF-nomogram showed good agreement with real observations in the training and validation sets, according to the calibration curve. The AIHF-nomogram performed significantly better than the fibrosis-4 and aminotransferase-to-platelet ratio scores in the training and validation sets, with an area under the ROCs for predicting advanced fibrosis of 0.804 in the training set and 0.781 in the validation set. DCA indicated that the AIHFI-nomogram was clinically useful. The nomogram will be available at http://ndth-zzy.shinyapps.io/AIHF-nomogram/as a web-based calculator.

**Conclusions:**

The novel, easy-to-use web-based AIHF-nomogram model provides an insightful and applicable tool to identify AIH patients with advanced liver fibrosis.

## Introduction

Autoimmune hepatitis (AIH) is an immune-mediated severe inflammatory liver disease characterized by the presence of interface hepatitis, elevated serum transaminase levels, hypergammaglobulinemia, and the presence of autoantibodies (1–3). Untreated patients tend to progress to end-stage liver disease with liver failure and the development of hepatocellular carcinoma (4). Currently, the main approach to treating AIH involves budesonide and azathioprine (AZA) or predniso(lo)ne and AZA as the first-line treatment (5). Glucocorticoids suppress the transcription of inflammatory genes and induce the transcription of immunosuppressive genes, while AZA may induce apoptosis of T cells, which exerts an immunosuppressive effect (6). A proper treatment regimen has demonstrated the ability to achieve and maintain disease remission, which not only stops the progression of fibrosis but also facilitates its regression, resulting in a favorable long-term prognosis (4). Therefore, monitoring liver fibrosis plays an important role in guiding treatment strategies and improving the prognosis of patients with AIH (7, 8).

Liver biopsy remains the gold standard for assessing liver fibrosis (9). However, a second or repeat liver biopsy cannot easily be performed due to the characteristics of this expensive and invasive diagnostic procedure (10). Therefore, the development of convenient and non-invasive tests (NITs) for evaluating liver fibrosis in AIH is urgently needed. NITs for liver fibrosis can be used at different steps for the better management of patients with AIH, such as the assessment of disease severity, evaluation of treatment response, decision on treatment withdrawal, and prediction of outcomes (11). Numerous NITs, including both laboratory and radiological tests, have been suggested for evaluating liver fibrosis. Laboratory-based tests such as the aspartate aminotransferase (AST)-to-platelet (PLT) ratio index (APRI) (12) and the fibrosis-4 index (FIB-4) (13) are simple, cost-effective, and widely available tests that have been extensively validated in various liver diseases (14, 15). However, the efficacy of these two NITs in detecting liver fibrosis in AIH patients is currently undetermined. Various studies have reported low diagnostic accuracy of APRI and FIB-4 in predicting liver fibrosis among AIH patients (16, 17). Transient elastography (TE) measurement is an established non-invasive radiological test used for liver fibrosis in various liver diseases and is based on the assessment of liver stiffness (18). Several studies have assessed the diagnostic efficacy and precision of TE, which has been found to exhibit superior performance in evaluating the stage of fibrosis in viral hepatitis patients when compared to other NITs such as APRI and FIB-4 (18, 19). Nevertheless, its clinical utility in AIH patients warrants further investigation, and the significance of TE in AIH patients is debatable, as the accuracy of TE in identifying liver fibrosis may be reduced by elevated serum alanine aminotransferase (ALT) levels (20). Furthermore, the high cost and technical requirements of TE may limit its application in clinical settings, especially in resource-limited regions (14). Although magnetic resonance elastography (MRE) can accurately detect advanced fibrosis in AIH, it requires special equipment and software (21, 22). Therefore, alternative non-invasive approaches to assessing liver fibrosis in patients with AIH need to be explored.

In this study, we intended to establish and validate an easy-to-use web-based nomogram that incorporated the conventional clinical indicators that can be easily obtained for predicting advanced liver fibrosis in AIH, as well as compare the predictive ability of the nomogram with APRI and FIB-4.

## Methods

### Patients

Consecutive patients with AIH who underwent liver biopsy between August 2011 and December 2021 were retrospectively included from five medical institutions, including Nanjing Drum Tower Hospital (Nanjing, China), The Second Hospital of Nanjing (Nanjing, China), The Affiliated Infectious Diseases Hospital of Soochow University (Suzhou, China), The Fifth People's Hospital of Wuxi (Wuxi, China), and Zhongda Hospital Southeast University (Nanjing, China). The diagnosis of AIH was made according to clinical, biochemical, serological, and histopathological findings consistent with clinical practice guidelines (8, 23). The exclusion criteria were co-infected with viral hepatitis or Epstein–Barr virus or concurrent with drug-induced liver injury, nonalcoholic fatty liver disease (NAFLD), primary biliary cirrhosis, primary sclerosing cholangitis, or alcoholic liver disease. The study was carried out in accordance with the Declaration of Helsinki’s ethical principles and was authorized by the Ethics Committee of the local hospital.

### Data acquisition

All patients’ medical records who participated in the study were collected retrospectively. Data on patients with AIH on demographics, clinical manifestations, laboratory tests, and histological reports were collected using a unified data frame. APRI and FIB-4 were calculated according to the following formulae: [AST (U/L)/upper limit of normal (ULN) of AST]/PLT (10^9^/L) × 100 for APRI and [age (years) × AST (U/L)]/[PLT (10^9^/L) × (ALT [U/L])^1/2^] for FIB-4 (12, 13).

### Liver histological assessment

All selected patients underwent an ultrasound-guided liver biopsy of at least one centimeter in length, with at least six accessible portal tracts. Each collected sample was evaluated by two experienced pathologists. According to the Scheuer grading system, liver fibrosis was classified as S0, no fibrosis; S1, portal fibrosis without septa; S2, portal fibrosis with rare septa; S3, numerous septa without cirrhosis; and S4, cirrhosis (24). Advanced liver fibrosis was defined as stage S ≥3 (25–27).

### Statistical analysis

Data management and analysis were performed in R version 4.2.0 (R Foundation, Vienna, Austria; https://www.r-project.org/). Data were presented as the median and interquartile range (IQR) of continuous variables or as the frequency (percentage) of categorical variables. Differences between groups of patients were detected using the Student’s t-test, Mann–Whitney U test, or Chi-square test. Statistical significance was defined as P <0.05. The training set and validation set were generated by the “sample” function in R. In the training set, least absolute shrinkage and selection operator (LASSO) regression was used to select predictors for evaluating advanced fibrosis in AIH. The selected predictors were introduced into a multivariate logistic regression, and the results were used to construct a nomogram. The web-based dynamic nomogram was conducted using the “shiny” package. The correlations between NIT scores and liver fibrosis stages were analyzed by Spearman rank correlation analysis. We evaluated the nomogram’s performance in terms of discrimination, calibration, and clinical usefulness. Model discrimination was evaluated using the areas under the receiver operating characteristic curve (AUROC). The calibration curve and Hosmer–Lemeshow goodness of fit test were used to assess model calibration. Decision curve analysis (DCA) was used to assess clinical usefulness.

## Results

### Clinical characteristics of patients

A total of 235 patients with AIH met the inclusion criteria and were eligible for this study. The flow chart for patient selection is presented in **Figure S1**. Baseline characteristics of patients are shown in **Table 1**. The age distribution of AIH patients was shown at all ages (**Figure S2**). The median age of patients was 54.0 (IQR: 46.0, 62.0) years old, and 195 (83.0%) patients were female. The median levels of ALT, immunoglobulin G (IgG), red cell distribution width (RDW), and PLT were 84.2 (IQR: 39.2, 203.5) U/L, 16.3 (IQR: 12.9, 20.2) g/L, 13.9 (IQR: 13.0, 15.4)%, and 150.0 (IQR: 107.5, 189.0) ×10^9^/L, respectively. Of all the patients with available data on antibodies, 147 were positive for anti-nuclear antibodies; 11 were positive for anti-smooth muscle antibodies; one was positive for anti-liver kidney microsomes type 1 antibodies; and seven were positive for anti-liver cytosol type 1 antibodies. The distributions of each stage of liver fibrosis were as follows: S0–1, 47 (20.0%) patients; S2, 66 (28.1%) patients; S3, 57 (24.2%) patients; and S4, 65 (27.7%) patients. The proportion of cirrhosis was significantly higher in elderly patients (≥65 years) compared to those under 65 years old (43.5% *vs*. 23.8%, P = 0.002) (**Table S1**).

**Table 1:** Characteristics for patients with autoimmune hepatitis.

Variables	Total (n = 235)	Training set (n = 157)	Validation set (n = 78)	P-value
Age (yr)	54.0 (46.0, 62.0)	53.0 (47.0, 60.0)	55.0 (46.0, 65.8)	0.205
Female (%)	195 (83.0)	130 (82.8)	65 (83.3)	0.919
RDW (%)	13.9 (13.0, 15.4)	14.0 (13.0, 15.3)	13.8 (13.0, 15.4)	0.754
PLT (×10^9^/L)	150.0 (107.5, 189.0)	151.0 (107.0, 188.0)	150.0 (108.2, 192.8)	0.797
TB (μmol/L)	21.7 (13.2, 41.9)	21.0 (13.0, 38.2)	24.6 (14.5, 48.0)	0.376
ALB (g/L)	37.7 (34.1, 40.2)	37.6 (34.1, 40.2)	37.7 (33.9, 40.2)	0.874
GLB (g/L)	30.9 (26.4, 36.2)	30.7 (26.4, 36.5)	31.6 (26.8, 35.4)	0.981
ALT (U/L)	84.2 (39.2, 203.5)	85.0 (39.4, 189.8)	80.2 (37.9, 228.5)	0.680
AST (U/L)	69.0 (39.8, 156.5)	65.5 (37.0, 130.0)	80.0 (46.2, 214.0)	0.089
ALP (U/L)	119.0 (87.0, 182.6)	116.4 (86.0, 187.7)	120.2 (91.2, 173.3)	0.780
GGT (U/L)	129.3 (62.0, 224.7)	131.0 (60.0, 231.0)	123.7 (65.5, 219.8)	0.656
PT (s)	13.1 (12.2, 14.1)	13.0 (12.2, 14.1)	13.3 (12.4, 14.1)	0.268
IgG (g/L)	16.3 (12.9, 20.2)	16.5 (12.8, 20.5)	15.9 (13.1, 19.4)	0.994
ANA (+)	147/226 (65.0)	103/150 (68.7)	44/76 (57.9)	0.145
SMA (+)	11/119 (9.2)	7/78 (9.0)	4/41 (9.8)	>0.99
LKM1 (+)	1/164 (0.6)	0/107 (0.0)	1/57 (1.8)	0.748
LC1 (+)	7/160 (4.4)	6/105 (5.7)	1/55 (1.8)	0.461
**Fibrosis stages (%)**				0.369
S0–1	47 (20.0)	34 (21.7)	13 (16.6)	
S2	66 (28.1)	42 (26.7)	24 (30.8)	
S3	57 (24.2)	34 (21.7)	23 (29.5)	
S4	65 (27.7)	47 (29.9)	18 (23.1)	

ALB, albumin; ALP, alkaline phosphatase; ALT, alanine aminotransferase; ANA, anti-nuclear antibodies; AST, aspartate aminotransferase; GGT, gamma-glutamyl transferase; GLB, globulin; IgG, immunoglobulin G; LC1, anti-liver cytosol type 1 antibodies; LKM1, anti-liver kidney microsomes type 1 antibodies; PLT, platelets; PT, prothrombin time; RDW, red cell distribution width; SMA, anti-smooth muscle antibodies; TB, total bilirubin.

In a 2:1 ratio, the patients were randomized into two sets: a training set and a validation set. There were no significant differences in clinical characteristics between the training set and validation set (**Table 1**).

### Comparison of clinical features of patients with and without advanced fibrosis

The training set included 51.6% of patients with advanced liver fibrosis (**Table S2**). As shown in **Table S2**, patients with advanced liver fibrosis were older (54.0 *vs*. 52.0, P = 0.035) and had higher median levels of total bilirubin (TB, 25.5 μmol/L *vs*. 16.2 μmol/L, P = 0.008), RDW (14.0% *vs*. 13.6%, P = 0.023), and prothrombin time (PT, 13.5s *vs*. 12.4s, P <0.001) compared to patients with non-advanced fibrosis, while lower median levels of albumin (ALB, 36.7 g/L *vs*. 38.8 g/L, P = 0.003) and PLT (132.0 × 10^9^/L *vs*. 177.0 × 10^9^/L, P <0.001) were found.

### Development of a nomogram for predicting advanced liver fibrosis

**Figure 1:**
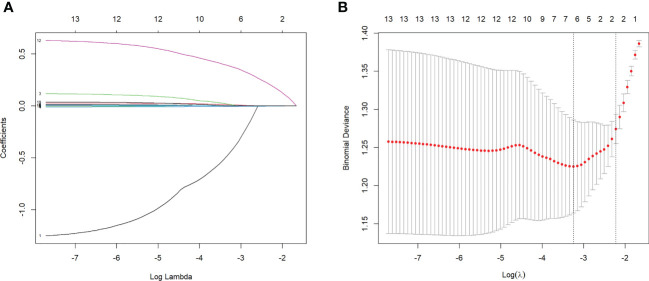
Selection of predictors by the LASSO regression. **(A)** LASSO coefficient profiles of clinical variables. **(B)** Identification of the optimal lambda in the LASSO regression analysis used 10-fold cross-validation *via* minimum criteria.

**Figure 2:**
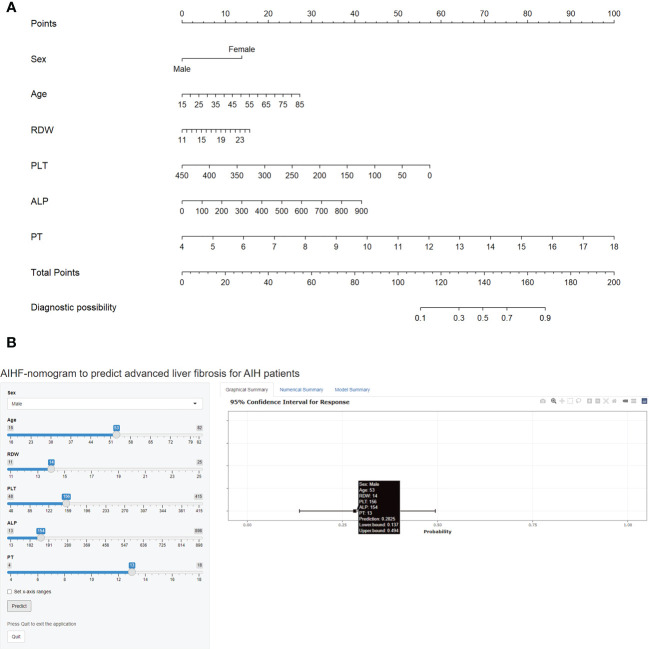
AIHF-nomogram for the prediction of advanced liver fibrosis in patients with autoimmune hepatitis. **(A)** AIHF-nomogram for the prediction of advanced liver fibrosis. **(B)** A screenshot of the web-based nomogram, which is accessible at http://ndth-zzy.shinyapps.io/AIHF-nomogram/. For example, a 53-year-old male AIH patient with an RDW of 14%, PLT of 156 × 10^9^/L, ALP of 154 U/L, and PT of 13 s has a diagnosed probability of advanced liver fibrosis of 28.3%.

For the predictors’ selection, using LASSO regression analysis, six most predictive variables with non-coefficients from 13 variables were selected *via* 10-fold cross-validation, including sex, age, RDW, PLT, alkaline phosphatase (ALP), and PT (**Figure 1**). These predictors were incorporated into the logistic regression model and then represented as an easy-to-use autoimmune hepatitis fibrosis (AIHF)-nomogram, which is available online (https://ndth-zzy.shinyapps.io/AIHF-nomogram/), as screenshotted in **Figure 2**.

**Figure 3:**
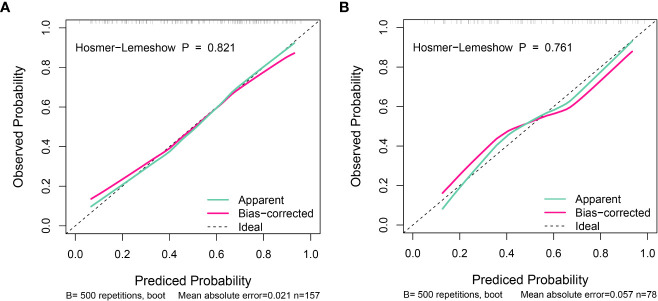
Calibration curves of the AIHF-nomogram for predicting advanced liver fibrosis in the training set **(A)** and validation set **(B)**.

**Figure 4:**
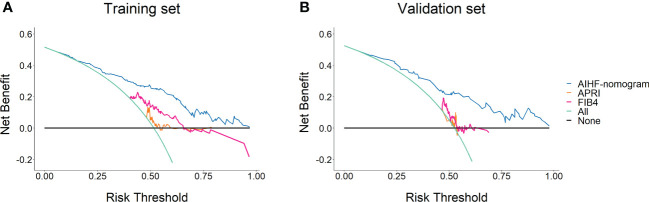
The decision curve analysis of the AIHF-nomogram for predicting advanced liver fibrosis in the training set **(A)** and validation set **(B)**.

The calibration curve was used to evaluate this predictive model (**Figure 3**). The 500-time bootstrapped curves indicated that AIHF-nomogram prediction differed slightly from actual observation, which showed good agreement between two datasets. Furthermore, the Hosmer–Lemeshow goodness of fit test demonstrated that the model fitted well in the training set (P = 0.821), and the validation set likewise demonstrated good accuracy (P = 0.761). The decision curve analysis for AIHF-nomogram is presented in **Figure 4**. The DCA demonstrated that the threshold probability of the model for predicting advanced fibrosis in AIH in the training set and validation set was 1.3%–96.8% and 5.9%–97.7%, respectively. The net benefit for patients using the model surpasses the treat-all scheme or the treat-none scheme, as well as the APRI and FIB-4 score strategies.

### Association of liver fibrosis stages with AIHF-nomogram, APRI, and FIB-4

We calculated the AIHF-nomogram scores in different liver fibrosis stages, which indicated an increasing tendency with liver fibrosis stages in the training set (P <0.001) and validation set (P <0.001). In comparison of APRI scores in AIH patients with different liver fibrosis stages, no statistical differences were detected in both the training (P = 0.099) and validation (P = 0.128) sets. There is a gradual increase in FIB-4 scores with liver fibrosis stages (P <0.001) in the training set, while no increasing trend was observed in the validation set (P = 0.213) (**Figure S3**).

Correlation analysis also indicated that the AIHF-nomogram scores were positively correlated with the fibrosis stages both in the training set (r = 0.58, P <0.001) and validation set (r = 0.55, P <0.001). The APRI scores were weakly correlated with the fibrosis stages in the training set (r = 0.19, P = 0.014), whereas no significant correlation was observed in the validation set (r = −0.14, P = 0.233). The FIB-4 scores were weakly correlated with the fibrosis stages in the training set (r = 0.38, P <0.001) but not in the validation set (r = 0.15, P = 0.180) (**Figure S4**).

### Comparisons among the AIHF-nomogram, APRI, and FIB-4 for predicting advanced fibrosis

The discrimination ability of the AIHF-nomogram was assessed using ROC analysis (**Figure S5**). The AUROC of the AIHF-nomogram in the training set was 0.804 (95% confidence interval [CI]: 0.735–0.872) with a sensitivity of 72.8% and a specificity of 78.9%, and the optimal cut-off value was 141.700. In the validation set, the AUROC of the AIHF-nomogram was 0.781 (95% CI: 0.676–0.887) with a sensitivity of 70.7% and a specificity of 75.7%, and the optimal cut-off value was 138.154. Pairwise comparison revealed that AIHF-nomogram was significantly better than APRI and FIB-4 in predicting advanced fibrosis in both sets, as shown in **Table 2**.

**Table 2:** Diagnostic performances of AIHF-nomogram, APRI, and FIB-4 in the training set and validation set.

	Training set	Validation set
AIHF-nomogram		
AUROC (95% CI)	0.804 (0.735–0.872)	0.781 (0.676–0.887)
Cutoff value	141.700	138.154
Sensitivity/specificity (%)	72.8/78.9	70.7/75.7
Accuracy (%)	75.8	73.1
PPV/NPV (%)	78.7/73.2	76.3/70.0
Positive/negative LR	3.460/0.344	2.908/0.387
APRI		
AUROC (95% CI)	0.592 (0.502–0.681)	0.467 (0.336–0.599)
Cutoff value	0.898	0.349
Sensitivity/specificity (%)	71.6/50.0	97.6/10.8
Accuracy (%)	61.1	56.4
PPV/NPV (%)	60.4/62.3	54.8/80.0
Positive/negative LR	1.432/0.568	1.094/0.226
P-value of ROC contrast test	<0.001	<0.001
FIB-4		
AUROC (95% CI)	0.683 (0.599–0.767)	0.587 (0.457–0.717)
Cutoff value	3.108	1.299
Sensitivity/specificity (%)	61.7/69.7	97.6/24.3
Accuracy (%)	65.6	62.8
PPV/NPV (%)	68.5/63.1	58.8/90.0
Positive/negative LR	2.040/0.549	1.289/0.100
P-value of ROC contrast test	<0.001	0.003

AUROC, area under the receiver operating characteristic curve; CI, confidence interval; LR, likelihood ratio; NPV, negative predictive value, PPV, positive predictive value.

## Discussion

Most patients with AIH were reported to have advanced fibrosis or even cirrhosis at the time of initial diagnosis due to the lack of specific diagnostic markers (28, 29). In this study, as many as 52.0% of patients with AIH were observed with advanced liver fibrosis, which indicated that a considerable proportion of the AIH patients had advanced liver disease when diagnosed. In cases where a diagnosis of AIH has been established, long-term treatment with corticosteroids alone or in combination with AZA is commonly recommended (28). The severity and distribution of fibrosis have been linked to disease progression and treatment response in patients with AIH (18). Thus, the identification of fibrosis at treatment onset and subsequent monitoring during long-term follow-up are of importance in clinical practice. Although liver biopsy remains the gold standard for assessing liver fibrosis, several shortcomings prevent it from being extensively used in clinical settings (7, 8, 10). APRI and FIB-4 have been widely used to evaluate liver fibrosis in patients with chronic hepatitis C and hepatitis B (30–33). However, the performance of these two NITs for diagnosing fibrosis in some AIH cohorts was limited (34, 35). A systematic review by Wu et al. (35) demonstrated that the accuracy of APRI and FIB-4 for staging liver fibrosis is poor. In our previous study, we also found that FIB-4 and APRI had limited ability to accurately identify advanced liver fibrosis in patients with autoimmune hepatitis (27). Consistently, the present study also found that the APRI and FIB-4 scores were not strongly correlated with the fibrosis stage, which showed an unsatisfactory performance in distinguishing advanced liver fibrosis in AIH patients. The good diagnostic accuracy of TE for advanced fibrosis and cirrhosis is only possible after 6 months of immunosuppressive therapy since TE estimates of liver stiffness are affected by both inflammation and fibrosis (36). Thus, the diagnostic accuracy of TE over different time periods should be interpreted with caution (36). Therefore, there is an urgent need to establish a NIT for the assessment of liver fibrosis severity in patients with AIH.

In the present study, we developed and validated a novel AIHF-nomogram containing sex, age, RDW, PLT, ALP, and PT, which showed good performance in predicting advanced liver fibrosis. There are several strengths to our study. The first is that we constructed a model by combining several simple and easily available relevant risk factors, then presented it as an easy-to-use web-based nomogram. The AIHF-nomogram is widely generalizable and will increase clinical efficiency, considering the high accuracy and discrimination revealed in the ROC curves. In the training set, the percent correctly classified was as high as 75.8% with an AUROC of 0.804, and in the validation set, it also had a high accuracy of 73.1% with an AUROC of 0.781. The training and validation sets showed consistent accuracy and better prediction performance for advanced liver fibrosis in AIH patients when compared to APRI and FIB-4. In addition, several methods were utilized to analyze the efficacy of the nomogram, with calibration curves demonstrating strong concordance between predicted and actual observations and DCA indicating the AIHF-nomogram’s high clinical applicability.

The sex, age, RDW, PLT, ALP, and PT indexes in this AIHF-nomogram were independent predictors of advanced liver fibrosis. Each of the predictors is routinely tested in clinical practice. In population studies of AIH, an almost universal feature is the predominance of women (37). It has been reported that 75%–80% of AIH patients were women, regardless of subtype (38). This characteristic was also corroborated by the fact that 83.1% of the AIH patients in our study were female. Consistent with the previous study, the age distribution of patients with AIH spans across all decades (5). A significant proportion (19.5%) of AIH patients in our study were elderly, which is consistent with the previous study (39). Moreover, elderly patients may progress to cirrhosis more rapidly than young adults with AIH (40). Our study also confirmed that the proportion of cirrhosis was significantly higher in elderly patients compared to those under 65 years old (43.5% *vs*. 23.8%, P = 0.002). Therefore, diagnosis and management of AIH in the elderly are important. However, the sample size of elderly patients in our study was limited (n = 24 in the training set and n = 22 in the validation set), which may have restricted further analysis of AIH in this population. The RDW is a measurement of the variability in red blood cell size, which is typically included in complete blood cell counts. Several studies indicated that RDW is related to the severity of chronic liver diseases (41–43). In some AIH cohorts, patients had elevated RDW levels (16, 44). RDW may be a promising biomarker of the severity of liver inflammation in patients with AIH, according to our previous studies, which demonstrated a positive correlation between RDW levels and the severity of liver inflammation in patients with AIH (45). PLT is essential for both the pathophysiology of fibrosis and liver regeneration (34). In our previous study, PLT was demonstrated to be associated with fibrosis in chronic hepatitis B (46). Numerous studies also demonstrated that decreased PLT counts correlated with fibrosis stages in patients with AIH, which is consistent with our present study that showed lower PLT counts in patients with advanced fibrosis compared to those with non-advanced fibrosis (17, 47). ALP is a hydrolase enzyme that is a common serologic test for liver function. Our previous study identified that ALP was significantly associated with fibrosis stages in CHB patients (46). Chang et al. (48) established a nomogram based on ALP to predict evident histological liver injury in patients with HBeAg-positive CHB. PT is prolonged by reduced coagulation factors and fibrinogen synthesis deficiency, suggesting the synthetic capacity of the hepatocytes is decreased with liver injury (49). PT prolongation is also an indicator of advanced liver fibrosis in AIH patients, as reported previously (47).

While our study established an AIHF-nomogram model with clinical significance for identifying AIH patients with advanced liver fibrosis, several limitations should be acknowledged. Firstly, as a retrospective study, caution is warranted in interpreting our results. In this study, we tried our best to include all eligible patients who received a liver biopsy and fulfilled our inclusion and exclusion criteria with sufficient data from the participating medical institutions during the study period. We acknowledge that the sample size of our study may be limited. In the future, our results will need to be validated in prospective, multi-center studies with a large sample size. However, we believe that the sample from five centers ensures the representativeness of our findings. Second, the Scheuer grading system instead of the METAVIR scoring system was used for staging liver fibrosis in the present study. Due to the discrepancy in scoring systems, the evaluation results for liver fibrosis might be different. Thus, more studies with other scoring systems, such as the METAVIR scoring system for staging fibrosis, are needed to validate our results. Thirdly, our study design was cross-sectional, and thus, the long-term prognostic value of the AIHF-nomogram requires confirmation in future research. Lastly, we were not able to compare the predictive performance for advanced liver fibrosis between the AIHF-nomogram and liver stiffness due to the small proportion of patients with liver stiffness data available.

In conclusion, the AIHF-nomogram is a valuable tool for predicting advanced liver fibrosis in AIH patients, utilizing readily available clinical parameters. Of particular note is the fact that this innovative model is accessible as a web-based dynamic nomogram, which is both convenient and user-friendly for physicians in clinical practice. This is especially important in resource-limited regions where access to more advanced diagnostic tools may be limited. Overall, the AIHF-nomogram represents a promising tool in the management of AIH patients and may help to improve clinical outcomes.

## Data availability statement

The data that support the study findings are available upon reasonable request from the corresponding authors (Rui Huang and Chao Wu).

## Ethics statement

The studies involving human participants were reviewed and approved by The Ethics Committee of Nanjing Drum Tower Hospital. Written informed consent for participation was not required for this study in accordance with the national legislation and the institutional requirements.

## Author contributions

All authors contributed to this study at different levels. All authors read and approved the final version. Study concept and design (CW, RH, and CZ). Acquisition of data (ZZ, JW, HW, YQ, LZ, JiaL, YC, YigL, YilL, YC, SY, XT, XY, YX, YY, QZ, and JieL). Statistical analysis and interpretation of data (ZZ, JW, and RH). Drafting of the manuscript (ZZ, JW, RH, HW, and YQ). Critical revision of the manuscript for important intellectual content (RH, CW, and CZ). All authors contributed to the article and approved the submitted version.
