# Supplementary material for: An easy-to-use AIHF-nomogram to predict advanced liver fibrosis in patients with autoimmune hepatitis
Source: Front Immunol. 2023 May 17;14:1130362. doi: 10.3389/fimmu.2023.1130362 (PMC10229817; doi:10.3389/fimmu.2023.1130362)
Supplement: Supplementary file 1 [file DataSheet_1.docx]

**Supplementary Material**

**An easy-to-use AIHF-nomogram to predict** **advanced liver fibrosis in patients with autoimmune hepatitis**

Zhiyi Zhang, Jian Wang, Huali Wang, Yuanwang Qiu, Li Zhu, Jiacheng Liu, Yun Chen, Yiguang Li, Yilin Liu, Yuxin Chen, Shengxia Yin, Xin Tong, Xiaomin Yan, Yali Xiong, Yongfeng Yang, Qun Zhang, Jie Li, Chuanwu Zhu, Chao Wu, Rui Huang

**Figure S1. Flow chart of patient selection.**


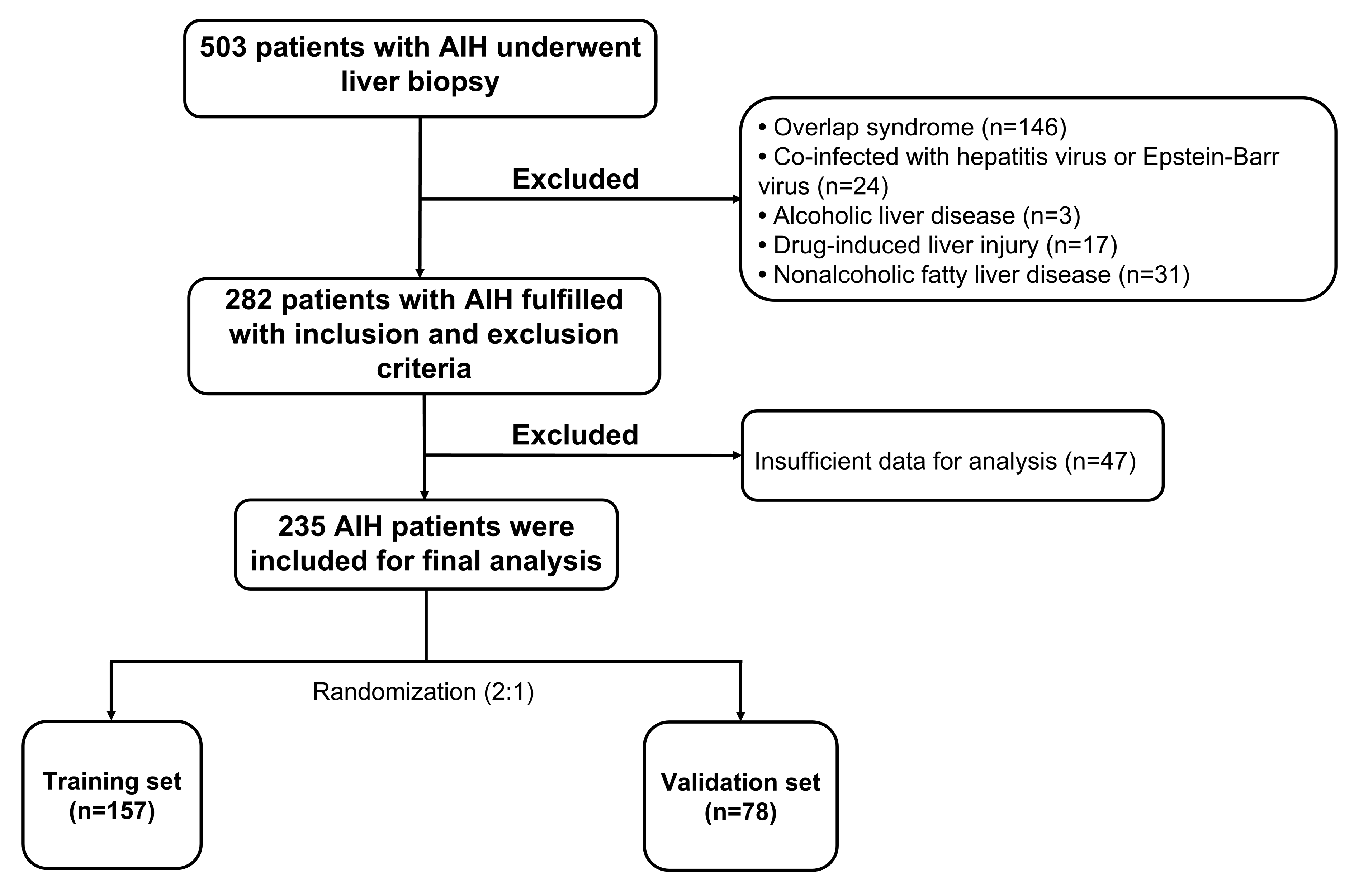


**Figure S2. Age distribution of patients with AIH.**


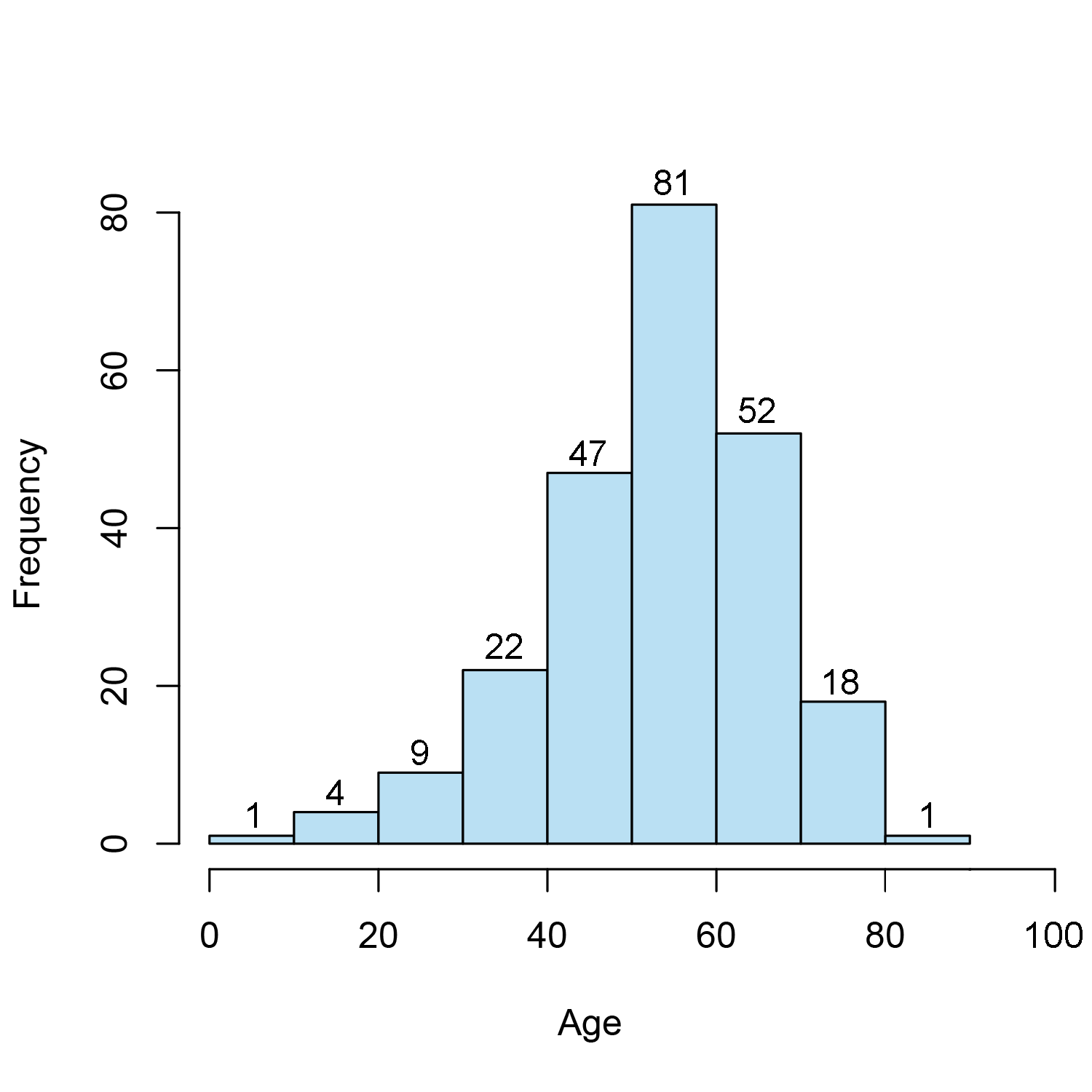


**Figure S3. The relationship between AIHF-nomogram scores,** **APRI scores, FIB-4 scores and liver fibrosis stages in the training and validation sets.**


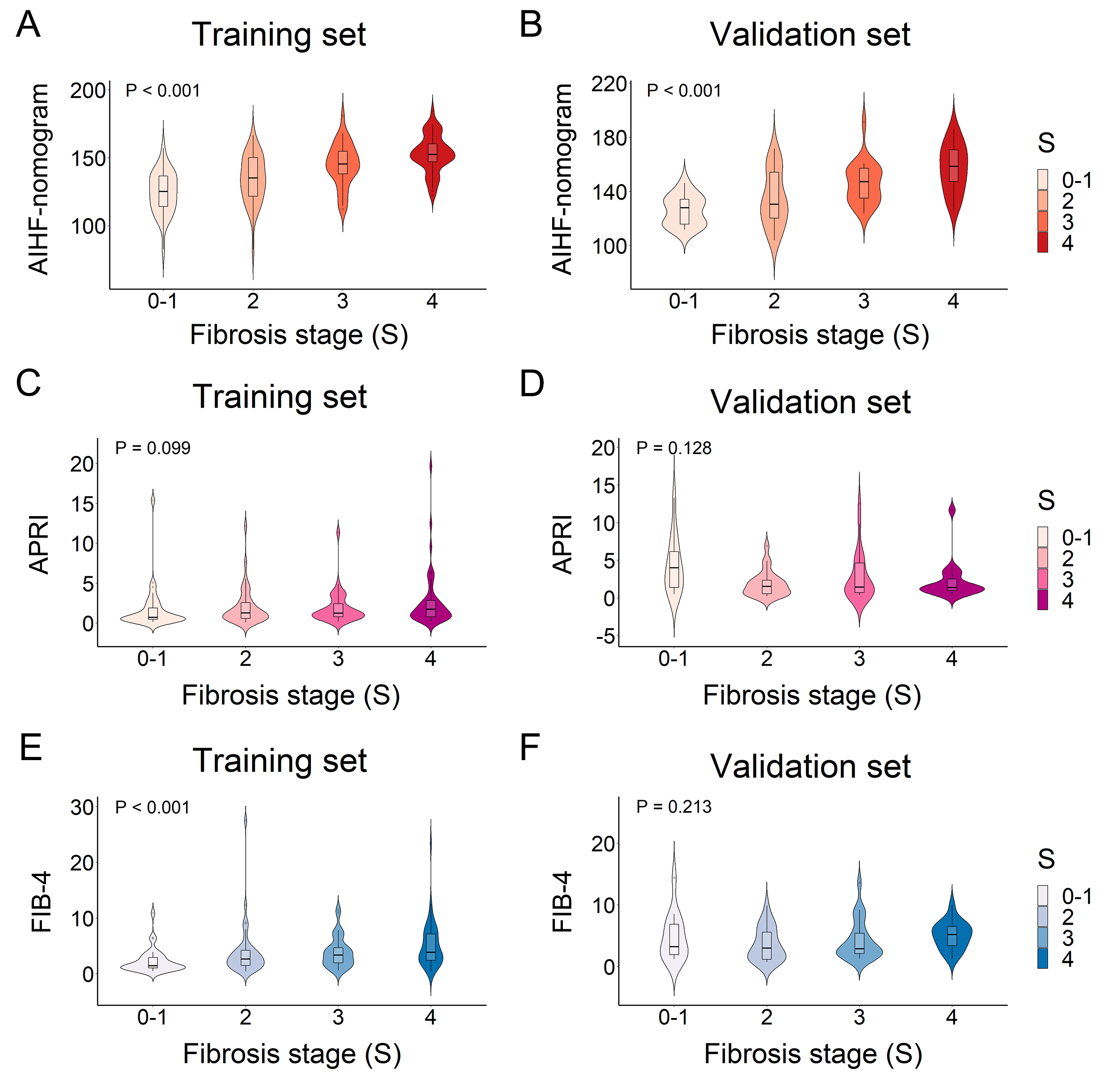


**Figure S4. Correlations between AIHF-nomogram scores, APRI scores, FIB-4 scores and liver fibrosis stages.** Training set (A) and validation set (B).


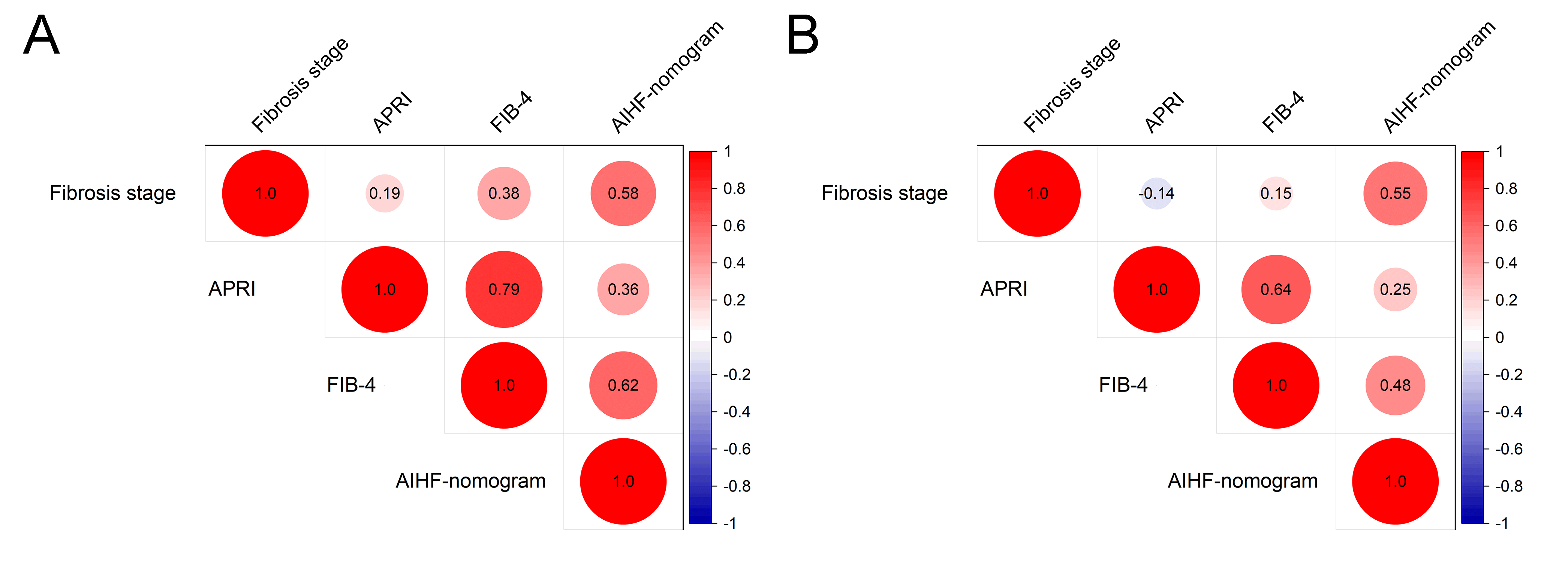


**Figure S5. Receiver operating characteristic curves for the prediction of advanced liver fibrosis in patients with autoimmune hepatitis.** Training set (A) and validation set (B).


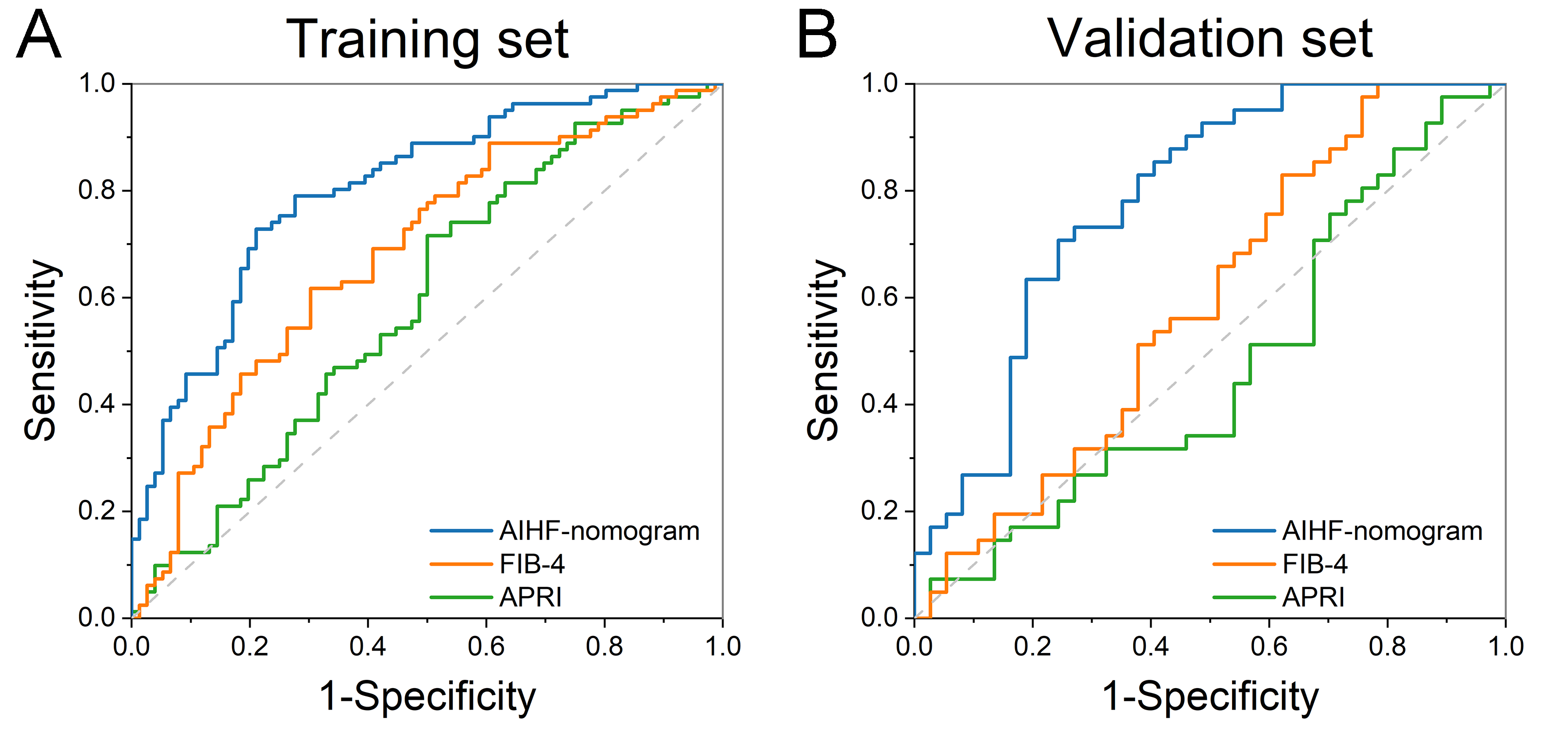


**Table S1. Comparisons of biochemical and clinical features of patients with autoimmune hepatitis according to the age at diagnosis.**

| **Variables** | **Total (n=235)** | **Age < 65 years (n = 189)** | **Age ≥ 65 years (n = 46)** | **P value** |
| --- | --- | --- | --- | --- |
| Age (yr) | 54.0 (46.0, 62.0) | 52.0 (44.0, 57.0) | 70.0 (67.2, 72.0) | <0.001 |
| Female (%) | 195 (83.0) | 161 (85.2) | 34 (73.9) | 0.081 |
| RDW (%) | 13.9 (13.0, 15.4) | 13.9 (13.0, 15.4) | 13.9 (13.0, 15.7) | 0.517 |
| PLT (×10^9^/L) | 150.0 (107.5, 189.0) | 158.0 (113.0, 195.0) | 122.5 (104.0, 152.8) | 0.003 |
| TB (μmol/L) | 21.7 (13.2, 41.9) | 21.0 (12.8, 39.0) | 25.0 (15.3, 45.3) | 0.130 |
| ALB (g/L) | 37.7 (34.1, 40.2) | 38.0 (34.4, 40.2) | 36.0 (32.7, 40.1) | 0.063 |
| GLB (g/L) | 30.9 (26.4, 36.2) | 30.4 (26.6, 35.3) | 33.5 (26.1, 39.1) | 0.140 |
| ALT (U/L) | 84.2 (39.2, 203.5) | 86.0 (40.0, 204.3) | 60.5 (33.5, 199.0) | 0.310 |
| AST (U/L) | 69.0 (39.8, 156.5) | 71.7 (40.3, 183.9) | 54.5 (38.5, 135.8) | 0.263 |
| ALP (U/L) | 119.0 (87.0, 182.6) | 117.8 (84.0, 187.0) | 122.0 (96.7, 172.7) | 0.479 |
| GGT (U/L) | 129.3 (62.0, 224.7) | 131.0 (60.0, 224.0) | 127.2 (77.1, 224.0) | 0.985 |
| PT (s) | 13.1 (12.2, 14.1) | 12.9 (12.2, 14.1) | 13.6 (12.9, 14.1) | 0.043 |
| IgG (g/L) | 16.3 (12.9, 20.2) | 15.9 (12.6, 19.9) | 17.4 (15.1, 20.4) | 0.054 |
| ANA (+) | 147/226 (65.0) | 123/182 (67.6) | 24/44 (54.5) | 0.147 |
| SMA (+) | 11/119 (9.2) | 9/103 (8.7) | 2/16 (12.5) | 0.642 |
| LKM1 (+) | 1/164 (0.6) | 0/129 (0.0) | 1/35 (2.9) | 0.213 |
| LC1 (+) | 7/160 (4.4) | 5/125 (4.0) | 2/35 (5.7) | 0.648 |
| **Fibrosis stages (%)** |  |  |  | 0.002 |
| S0-1 | 47 (20.0) | 46 (24.3) | 1 (2.2) |  |
| S2 | 66 (28.1) | 54 (28.6) | 12 (26.1) |  |
| S3 | 57 (24.3) | 44 (23.3) | 13 (28.3) |  |
| S4 | 65 (27.7) | 45 (23.8) | 20 (43.5) |  |

ALB, albumin; ALP, alkaline phosphatase; ALT, alanine aminotransferase; ANA, anti-nuclear antibodies; AST, aspartate aminotransferase; GGT, gamma-glutamyl transferase; GLB, globulin; IgG, immunoglobulin G; LC1, anti-liver cytosol type 1 antibodies; LKM1, anti-liver kidney microsomes type 1 antibodies; PLT, platelets; PT, prothrombin time; RDW, red cell distribution width; SMA, anti-smooth muscle antibodies; TB, total bilirubin.

**Table S2. Comparisons of biochemical and clinical features of patients with autoimmune hepatitis with and without advanced liver fibrosis.**

| **Variables**  **(n (%) or median (IQR))** | **Training set** | | |  | **Validation set** | | |
| --- | --- | --- | --- | --- | --- | --- | --- |
|  | **Advanced fibrosis**  **(n = 81)** | **Non-advanced fibrosis**  **(n = 76)** | **P value** |  | **Advanced fibrosis**  **(n = 41)** | **Non-advanced fibrosis**  **(n = 37)** | **P value** |
| Age (yr) | 54.0 (48.0, 64.0) | 52.0 (43.8, 58.2) | 0.035 |  | 63.0 (45.0, 69.0) | 53.0 (46.0, 60.0) | 0.050 |
| Female (%) | 71 (87.7) | 59 (77.6) | 0.147 |  | 34 (82.9) | 31 (83.8) | 0.919 |
| RDW (%) | 14.0 (13.3, 15.8) | 13.6 (12.8, 14.9) | 0.023 |  | 14.3 (13.2, 15.7) | 13.2 (12.5, 14.6) | 0.010 |
| PLT (×10^9^/L) | 132.0 (91.0,162.0) | 177.0 (135.0, 215.5) | <0.001 |  | 120.0 (104.0,171.0) | 176.0 (126.0, 229.0) | 0.002 |
| TB (μmol/L) | 25.5 (16.4, 44.0) | 16.2 (12.0, 33.4) | 0.008 |  | 25.4 (15.6, 45.8) | 22.0 (12.4, 48.7) | 0.450 |
| ALB (g/L) | 36.7 (32.8, 38.9) | 38.8 (35.7, 40.6) | 0.003 |  | 36.0 (32.7, 39.1) | 38.9 (36.1, 40.7) | 0.015 |
| GLB (g/L) | 31.9 (27.0, 38.2) | 29.2 (26.4, 34.9) | 0.080 |  | 33.7 (30.4, 36.4) | 28.1 (26.0, 32.9) | 0.002 |
| ALT (U/L) | 70.0 (38.0, 152.0) | 97.2 (46.0, 192.6) | 0.232 |  | 54.6 (28.7, 185.1) | 114.6 (56.4, 329.1) | 0.013 |
| AST (U/L) | 65.0 (38.0, 129.0) | 67.5 (36.2, 138.2) | 0.918 |  | 61.0 (43.5, 139.6) | 131.2 (53.6, 292.0) | 0.083 |
| ALP (U/L) | 116.4 (84.0, 192.1) | 117.6 (87.9, 154.7) | 0.506 |  | 117.8 (93.0, 173.0) | 120.7 (79.5, 173.4) | 0.589 |
| GGT (U/L) | 128.0 (74.7, 243.1) | 143.5 (53.1, 220.2) | 0.920 |  | 110.0 (61.0, 202.4) | 141.1 (82.8, 229.0) | 0.293 |
| PT (s) | 13.5 (12.8, 14.7) | 12.4 (11.7, 13.4) | <0.001 |  | 13.9 (13.0, 14.8) | 12.6 (11.8, 13.3) | <0.001 |
| IgG (g/L) | 17.0 (13.1, 20.4) | 15.4 (12.4, 20.7) | 0.285 |  | 17.0 (14.1, 20.9) | 15.5 (12.9,18.7) | 0.211 |

ALB, albumin; ALP, alkaline phosphatase; ALT, alanine aminotransferase; AST, aspartate aminotransferase; GGT, gamma-glutamyl transferase; GLB, globulin; IgG, immunoglobulin G; PLT, platelets; PT, prothrombin time; RDW, red cell distribution width; TB, total bilirubin.
